# Anxiety is associated with higher recurrence of atrial fibrillation after catheter ablation: A meta‐analysis

**DOI:** 10.1002/clc.23753

**Published:** 2022-01-18

**Authors:** Hong Du, Lei Yang, Zheng Hu, Hui Zhang

**Affiliations:** ^1^ Department of Cardiology Second Hospital of Hebei Medical University Shijiazhuang China; ^2^ Department of Neurosurgery Shijiazhuang People's Hospital Shijiazhuang China

**Keywords:** anxiety, atrial fibrillation, catheter ablation, meta‐analysis, recurrence

## Abstract

Previous studies that evaluated the influence of anxiety on recurrence of atrial fibrillation (AF) after catheter ablation showed inconsistent results. We performed a meta‐analysis of cohort study to systematically evaluate the association between anxiety and AF recurrence after catheter ablation. Electronic databases of PubMed, Embase, and Web of Science were searched for relevant cohort studies from inception to January 20, 2021. We applied the random‐effect model to combine the results to incorporate the potential influence of heterogeneity among studies. Five cohort studies were eligible for the meta‐analysis, which included 549 patients with AF that received catheter ablation. No significant heterogeneity was observed among the included studies (*I* ^2^ = 7%, *P* for Cochrane's *Q* test = 0.37). During a mean follow‐up of 9.7 months, 216 (39.3%) cases of recurrent AF occurred. Results of the meta‐analysis showed that anxiety was independently associated with an increased risk of AF recurrence after catheter ablation (adjusted relative risk: 2.36, 95% confidence interval: 1.71–3.26; *p* < .001). Subgroup analyses did not show that differences in study characteristics including study design, ethnicity of the patients, sample size, AF type, anxiety evaluation method, follow‐up duration, or adjustment of LAD may significantly affect the association between anxiety and AF recurrence (*p* for subgroup difference all > .10). Anxiety may be an independent risk factor for AF recurrence after catheter ablation. Whether alleviating anxiety mood could reduce the risk of AF recurrence after catheter ablation should also be investigated.

## 1 INTRODUCTION

Atrial fibrillation (AF) is one of the most prevalent arrhythmias, particularly in the elderly population.^1^, ^2^, ^3^ Patients with AF are at higher risk of many adverse clinical outcomes, such as arterial thromboembolism, heart failure, and mortality.^4^ With the acceleration of global aging, number of patients with AF is expected to grow rapidly, which highlights the importance of optimized management of the disease.^5^ Catheter ablation has now been recognized as an effective treatment for AF patients.^6^ Current international guidelines for the management of AF recommend catheter ablation for AF patients because it is associated with improved quality of life (QoL), as well as prognosis in AF patients.^7^, ^8^ However, substantial patients suffer from AF recurrence after catheter ablation, which limited its extensive use in clinical practice.^9^, ^10^ Anxiety is a common affective disorder in patients with cardiovascular diseases.^11^ Previous studies showed that an anxiety mood in patients with various cardiovascular conditions is related to the incidence of poor clinical outcomes, such as in patients with myocardial infarction,^12^ those after percutaneous coronary intervention,^13^ and subjects after cardiac surgeries.^14^ However, the association between anxiety and AF remains not fully determined.^15^ Previous meta‐analyses did not support a significant association between anxiety and an increased risk of AF in the general population.^16^, ^17^ Moreover, studies that evaluated the association between anxiety and AF recurrence after catheter also showed inconsistent results.^18^, ^19^, ^20^, ^21^, ^22^ This is possibly because most of them were small‐scale stuides^18^, ^19^, ^20^ (sample size < 100) which may be statistically inadequate to indicate a significant association between anxiety and AF recurrence. Therefore, in this study, we performed a meta‐analysis of cohort studies to systematically evaluate the association between anxiety and the risk of AF recurrence after catheter ablation. Besides, potential influences of patient and study characteristics on this association were also analyzed.

## 2 METHODS

This meta‐analysis was designed, conducted, and reported in accordance with MOOSE (Meta‐analysis of Observational Studies in Epidemiology)^23^ and Cochrane's Handbook^24^ guidelines.

### 2.1 Literature search

Electronic databases of PubMed, Embase, and Web of Science were searched for relevant cohort studies from inception to January 20, 2021. We applied a combined search term of “anxiety” OR “tension” OR “posttraumatic stress disorder” OR “panic” OR “phobia” OR “phobic” OR “worry,” with “atrial fibrillation” OR “AF,” and “ablation” OR “catheter” OR “pulmonary vein isolation” OR “PVI” OR “recurrence.” We only considered studies published in English. As a complementation, we also searched the reference lists of the related original and review articles for potential relevant studies.

### 2.2 Inclusion and exclusion criteria

Studies that fulfilled all of the criteria were considered to be eligible for the meta‐analysis: (1) designed as cohort studies; (2) patients with AF that were scheduled for catheter ablation were included; (3) anxiety was evaluated for the AF patients before the ablation procedure and considered as exposure; (4) documented the incidence of AF recurrence during follow‐up in AF patients with and without anxiety; (5) reported the multivariate‐adjusted risk ratios (RRs) for the recurrence of AF in patients with anxiety as compared to those without anxiety, at least for age and gender. Anxiety was evaluated and validated in accordance with the criteria applied in the original articles. We did not apply restrictions of minimal sample sizes or follow‐up durations for the potentially included studies. For repeated reports of the same cohort, latest studies with the longest follow‐up duration were included. For studies reported RRs of different adjusting levels, the most adequately adjusted data were extracted.

### 2.3 Data extracting and quality evaluation

Two authors (H. D. and L. Y.) performed database search, data extraction, and quality assessment independently. If discrepancies occurred, consultation with the third author (Z. H.) was indicated to resolve them. The following data regarding study characteristics were recorded: (1) study information (first author, publication year, design and location); (2) patient characteristics (age and sex of the included patients, and clinical classification of AF); (3) methods for the evaluation and validation of anxiety; (4) details of catheter ablation procedures; (5) follow‐up durations; and (6) methods for the validation of the AF recurrence outcome. We evaluated the quality of the included studies using the Newcastle–Ottawa Scale.^25^ This system rated the quality of cohort study based on items of three domains, including study group selection, between‐group comparability, and strategy for outcome validation.

### 2.4 Statistical analyses

Data of RRs and their corresponding standard errors were calculated from 95% confidence intervals (CIs) or *p* values, and were logarithmically transformed to stabilize variance and normalized the distribution.^24^ If hazard ratios were reported among the included studies, they were treated as RRs directly. Where the odds ratios (ORs) were presented, data were converted to RRs for the meta‐analysis (RR = OR/([1 − pRef] + [pRef × OR]), where pRef is the prevalence of the outcome in the reference group (nonanxiety group) as indicated by previous studies.^26^ The Cochrane's *Q* test and *I* ^2^ test were performed to evaluate the heterogeneity among studies.^27^ An *I* ^2^ > 50% indicates significant heterogeneity. A random‐effect model was applied for the meta‐analysis since it incorporates the potential influence of heterogeneity and thereby could retrieve a more generalizable result.^24^ Sensitivity analyses by removing individual study one at a time were performed to evaluate the stability of the results.^28^ Subgroup analyses were performed to evaluate the potential influences of study characteristics on the outcome, including patient ethnicity, study design, patient number, type of AF, methods for anxiety evaluation, follow‐up duration, and adjustment of left atrial dimension (LAD). Medians of the continuous variables were used as cut‐off values for stratification. Potential publication bias was assessed by funnel plots with the Egger regression asymmetry test.^29^ A *p* < .05 was considered as statistically significant. RevMan (Version 5.1; Cochrane Collaboration) and STATA software (Version 12.0; Stata Corporation) were used for the statistical analyses.

## 3 RESULTS

### 3.1 Results of database search and study inclusion

**Figure 1:**
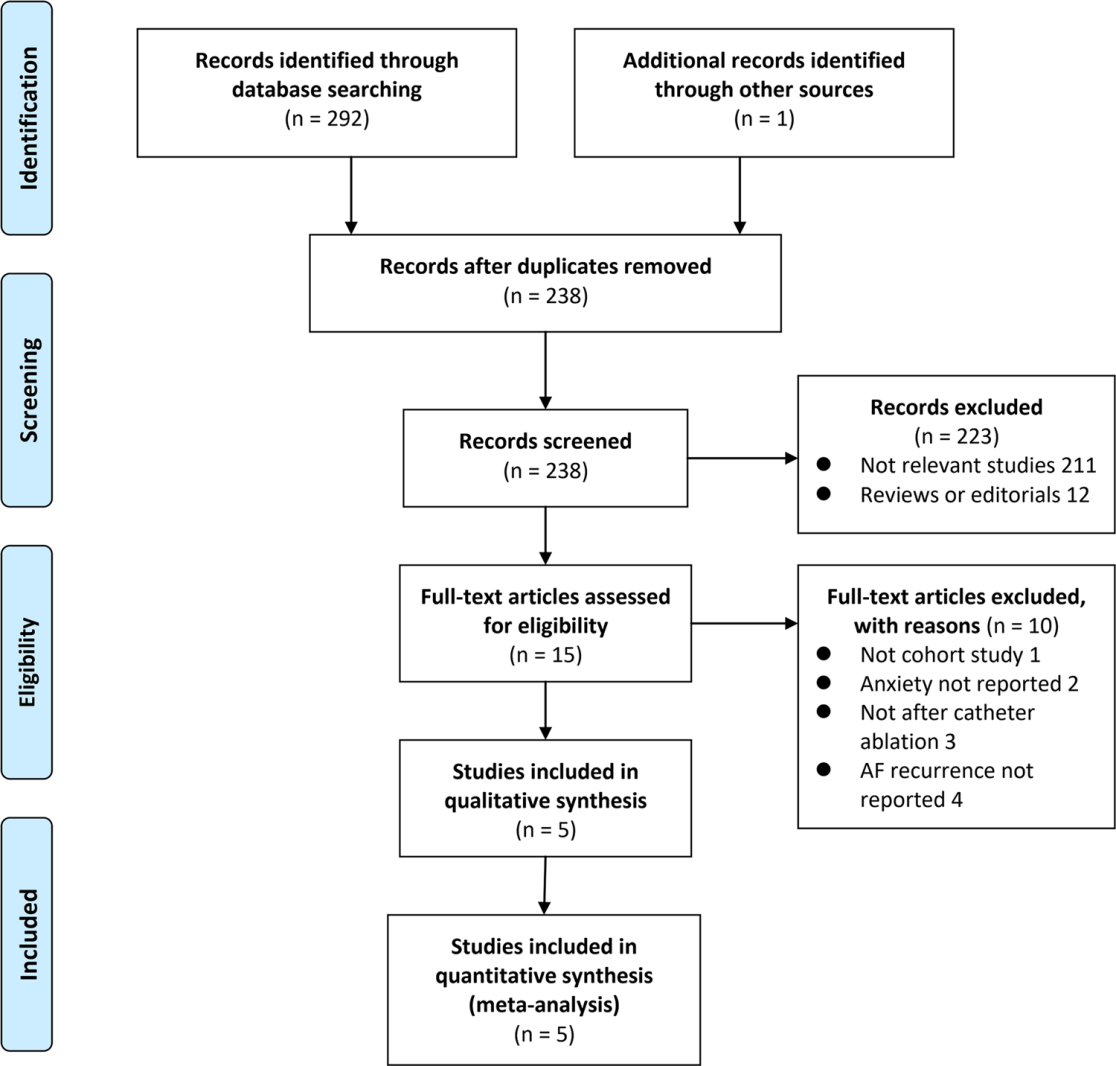
Preferred Reporting Items for Systematic Reviews and Meta‐Analyses flowchart of literature search

The processes of database search and study identification were presented in Figure 1. Briefly, 238 studies were retrieved via initial literature search after excluding duplications, and 223 were further excluded based on reading titles and abstracts primarily because they were irrelevant to the purpose of current meta‐analysis. Full‐text review involved the remaining 15 studies. Of them, 10 studies were further excluded because of reasons listed in Figure 1. Finally, five cohort studies^18^, ^19^, ^20^, ^21^, ^22^ were included.

### 3.2 Study characteristics and quality evaluation

The characteristics of the included studies were presented in Table 1. Overall, five cohort studies from China, Korea, Greece, and the Netherlands including 549 patients with AF that underwent catheter ablation were included. Two of them were retrospective studies,^18^, ^19^ while the other three were prospective cohorts.^20^, ^21^, ^22^ The sample sizes of the included cohorts varied from 43 to 239. One study included patients with persistent AF,^19^ two studies included paroxysmal AF,^18^, ^20^ while the other two included both.^21^, ^22^ Anxiety was evaluated via Zung Self‐Rating Anxiety Scale,^18^, ^19^ the State‐Trait Anxiety Inventory,^20^, ^21^ and the Cardiac Anxiety Questionnaire^22^ among the included studies. Patients of all the included studies received circumferential pulmonary vein isolation as the ablation strategy for AF. The follow‐up duration varied between 3 and 12 months. Events of AF recurrence that last at least for 30 s were validated by the clinical evaluation with electrocardiograph and Holter examinations. The NOS of the included studies ranged between seven and nine stars (Table 2), suggesting good study quality.

**Table 1:** Overview of the included cohort studies

Study	Country	Design	Sample size	Clinical classification of AF	Mean age (years)	Male proportion (%)	Anxiety validation	Ablation procedure	Follow‐up duration (months)	AF recurrence validation	Number of patients with AF recurrence	Adjustment of variables
Yu (2012a)	China	RC	43	Persistent	58.3	67.4	SAS	CPVI	12	ECG and Holter	17	Age, sex, AF duration, and LAD
Yu (2012b)	China	RC	98	Paroxysmal	55.2	50	SAS	CPVI	12	ECG and Holter	28	Age, sex, MR, TR, and LAD
Efremidis (2014)	Greece	PC	57	Paroxysmal	56.9	57.6	STAI	CPVI	8	ECG and Holter	16	Age, sex, BMI, HTN, DM
Jeon (2017)	Korea	PC	239	Paroxysmal and persistent	55.7	80.9	STAI	CPVI	12	ECG and Holter	139	Age, sex, BMI, CHADS2 Score, comorbidities, LAD, and AF classification
Knobel (2019)	Netherlands	PC	112	Paroxysmal and persistent	61.2	68	CAQ	CPVI	3	ECG and Holter	16	Age and sex

Abbreviations: AF, atrial fibrillation; CAQ, Cardiac Anxiety Questionnaire; ECG, electrocardiogram; LAD, left atrial dimension; MR, mitral regurgitation; PC, prospective cohort; RC, retrospective cohort; SDS, Zung Self‐Rating Anxiety Scale; STAI, the State‐Trait Anxiety Inventory; TR, tricuspid regurgitation.

**Table 2:** Study quality evaluation by the Newcastle–Ottawa Scale

Study	Representativeness of the exposed cohort	Selection of the nonexposed cohort	Ascertainment of exposure	Outcome not present at baseline	Control for age and sex	Control for other confounding factors	Assessment of outcome	Enough long follow‐up duration	Adequacy of follow‐up of cohorts	Total
Yu (2012a)	0	1	1	1	1	1	1	1	0	7
Yu (2012b)	0	1	1	1	1	1	1	1	0	7
Efremidis (2014)	0	1	1	1	1	0	1	1	1	7
Jeon (2017)	1	1	1	1	1	1	1	1	1	9
Knobel (2019)	1	1	1	1	1	0	1	0	1	7

### 3.3 Association between depression and recurrence of AF after catheter ablation

**Figure 2:**
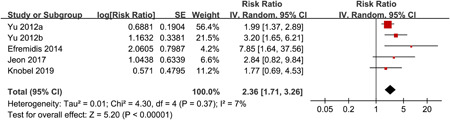
Forest plots for the meta‐analysis of the association between anxiety and AF recurrence after ablation. AF, atrial fibrillation; CI, confidence interval

During a mean follow‐up of 9.7 months, 216 (39.3%) patients had AF recurrence. No significant heterogeneity was observed among the included studies (*I* ^2^ = 7%, *P* for Cochrane's *Q* test = 0.37). During a mean follow‐up of 9.7 months, 216 (39.3%) cases of recurrent AF occurred. Results of the meta‐analysis showed that anxiety was independently associated with an increased risk of AF recurrence after catheter ablation (adjusted RR: 2.36, 95% CI: 1.71–3.26; *p* < .001; Figure 2). Sensitivity analyses omitting one study at a time retrieved similar results (RR: 2.17–2.94, *p*all < .001). Predefined subgroup analyses did not show that differences in study characteristics including study design, ethnicity of the patients, sample size, AF type, anxiety evaluation method, follow‐up duration, or adjustment of LAD may significantly affect the association between anxiety and AF recurrence (*p* for subgroup difference all > .10; Table 3).

**Table 3:** Subgroup analysis for the influence of anxiety on AF recurrence after ablation

	Datasets	RR (95% CI)	*p* for subgroup effect	*I* ^2^	*p* for subgroup difference
Ethnicity					
Asian	3	2.27 (1.65, 3.10)	<.001	0%	
Caucasian	2	2.63 (1.17, 5.88)	.02	61%	.74
Study design					
Retrospective	2	2.23 (1.61, 3.09)	<.001	33%	
Prospective	3	2.69 (1.37, 5.28)	<.001	22%	.63
Patient number					
≤100	3	2.35 (1.71, 3.23)	<.001	48%	
>100	2	2.10 (1.01, 4.45)	.04	0%	.79
AF clinical classification					
Persistent	1	1.99 (1.37, 2.89)	<.001	—	
Paroxysmal	2	3.67 (1.99, 6.75)	<.001	7%	
Paroxysmal or persistent	2	2.10 (1.01, 4.45)	.04	0%	.24
Anxiety evaluation					
SAS	2	2.23 (1.61, 3.09)	<.001	33%	
STAI	2	4.21 (1.59, 11.13)	.004	0%	
CAQ	1	1.77 (0.69, 4.53)	.23	—	.40
Follow‐up durations					
≤8 months	2	2.63 (1.17, 5.88)	.02	61%	
>8 months	3	2.27 (1.65, 3.10)	<.001	0%	.74
LAD adjusted					
Yes	3	2.27 (1.65, 3.10)	<.001	0%	
No	2	2.63 (1.17, 5.88)	.02	61%	.74

Abbreviations: AF, atrial fibrillation; CAQ, Cardiac Anxiety Questionnaire; CI, confidence interval; LAD, left atrial dimension; RR, risk ratio; SDS, Zung Self‐Rating Anxiety Scale; STAI, the State‐Trait Anxiety Inventory.

### 3.4 Publication bias

Funnel plots for the meta‐analysis of the association between anxiety before procedure and AF recurrence after catheter ablation were shown in Figure S1. The plots were symmetrical on visual inspection, indicating low risk of publication bias. The Egger's regression test was not performed because less than 10 datasets were included for the meta‐analysis.

## 4 DISCUSSION

In this meta‐analysis, by pooling the results of relevant cohort studies, we found that anxiety is independently associated with an increased risk of AF recurrence after catheter ablation. Further results by excluding one study at a time confirmed the stability of the findings, which was not driven by a certain included cohort. In addition, subgroup according to the study design, ethnicity of the patients, sample size, AF type, anxiety evaluation method, follow‐up duration, and adjustment of left atrial dimension did not show a significant influence on the association of interest, which further validated the robustness of the findings. Taken together, these results suggested that anxiety is an independent risk factor for AF recurrence after catheter ablation. These findings should be validated in large‐scale prospective studies. Moreover, in view of high incidence of AF recurrence after catheter ablation in current real‐world practice, potential effects of psychotherapy and anxiolytics on AF recurrence after catheter ablation in patients with anxiety should be evaluated.

To the best of our knowledge, this study is the first meta‐analysis that evaluated the association between anxiety and AF recurrence after catheter ablation. The strengths of the study include the strict inclusion criteria and comprehensive data analysis. Only cohort studies were included, which thereby could provide a temporary relationship between anxiety and AF recurrence. Besides, only data with multivariate adjustment were used, which could derive an independent association between anxiety and AF recurrence after catheter ablation. In addition, both sensitivity and subgroup analyses were used to confirm the robustness of the finding. Finally, no significant heterogeneity was found among the included cohort studies, which also reflected the consistency of the study design of the included cohorts. Previous studies also found that anxiety was independently associated with increased risks of early AF recurrence after cardioversion,^30^ as well as AF incidence after cardiac surgery.^31^ These findings, together with the findings from our meta‐analysis, may suggest that anxiety is a potential trigger of AF incidence or recurrence.^15^ It has been confirmed that anxiety is associated with low‐degree inflammatory response,^32^ increased sympathetic nerve activity,^33^ and activated neurohormonal pathways involved in atrial remodeling, such as the renin–angiotensin–aldosterone system.^34^ These pathophysiological changes have been confirmed to be involved in the pathogenesis of AF.^15^ Moreover, a recent cross‐sectional study showed that anxiety is independently associated with atrial cardiopathy in patients who were free of AF, atrial flutter, stroke, acute coronary syndrome and valvular heart disease, suggesting the triggering effect of anxiety on atrial remodeling.^35^ This association indicates the triggering effect of anxiety on atrial remodeling. Future studies are needed to determine the key molecular pathways underlying the association between anxiety and AF recurrence after catheter ablation.

Currently, it remains unknown whether interventions such as psychotherapy or anxiolytics could reduce the risk of AF recurrence in patients with anxiety. Interestingly, it has also been shown that anxiety is also associated with poor QoL in AF patients after catheter ablation,^36^ besides its adverse influence on AF recurrence. However, it does not mean that catheter ablation for AF should not be performed in people with anxiety, because it has been shown that in patients with primarily low‐burden paroxysmal AF, the reduction in AF burden following ablation may be associated with a clinically meaningful improvement in QoL, including relieved anxiety.^37^ In addition, a recent clinical trial in patients undergoing off‐pump coronary artery bypass graft showed that anxiety administrated by dexmedetomidine is associated with lower incidence of postoperative AF.^38^ From a clinical perspective, it could be hypothesized that interventions for relieving of anxiety mood during the perioperative and postoperative periods of AF catheter ablation may be effective in improving the QoL and reduce AF recurrence in these patients. Clinical studies may be considered in the future.

## 5 STUDY LIMITATIONS

Some limitations of our meta‐analysis should be mentioned. First, limited studies were included, particularly for the subgroup analysis. Accordingly, the results of subgroup analysis should be interpreted with caution. Second, the follow‐up durations of the included cohorts varied between 3 and 12 months, whether anxiety is associated with long‐term recurrence of AF after catheter ablation remains unknown. Third, various instruments were used for anxiety evaluation among the included studies. The optimal instruments for anxiety evaluation among these patients remain to be established. Moreover, although multivariate‐adjusted data were used, we could not exclude that some residual factors may confound the association between anxiety and increased risk of AF recurrence, such as concurrent use of statins.^39^ Besides, only full‐text article in peer‐reviewed journals were included to assure the reliability of the findings. However, excluding gray literature (such as conference abstract, unpublished data etc.) may increase the risk of publication bias. Finally, a causative association between anxiety and AF recurrence after catheter ablation could not be derived based on our study since it is a meta‐analysis of observational studies.

## 6 CONCLUSION

In conclusion, the results of this meta‐analysis showed that anxiety is an independent risk factor for AF recurrence after catheter ablation. These findings should be validated in large‐scale prospective studies, and the potential effects of psychotherapy and anxiolytics on AF recurrence after catheter ablation in patients with anxiety should be evaluated.

## CONFLICT OF INTERESTS

The authors declare that there are no conflict of interests.

## AUTHOR CONTRIBUTIONS

Hong Du and Lei Yang designed the study. Hong Du and Lei Yang performed database search, quality assessment, and data extraction. Hong Du, Hui Zhang, and Zheng Hu performed the statistical analysis. All authors interpreted the results. Hong Du and Zheng Hu drafted the manuscript. All authors critically revised the manuscript and approved the submission of the study.

## Supporting information

Supporting information.

## Data Availability

The data that support the findings of this study are available from the corresponding author upon reasonable request.
