# Supplementary material for: Anxiety is associated with higher recurrence of atrial fibrillation after catheter ablation: A meta‐analysis
Source: Clin Cardiol. 2022 Jan 18;45(3):243–50. doi: 10.1002/clc.23753 (PMC8922539; doi:10.1002/clc.23753)
Supplement: Supplementary file 1 — Supporting information. [file CLC-45-243-s001.docx]

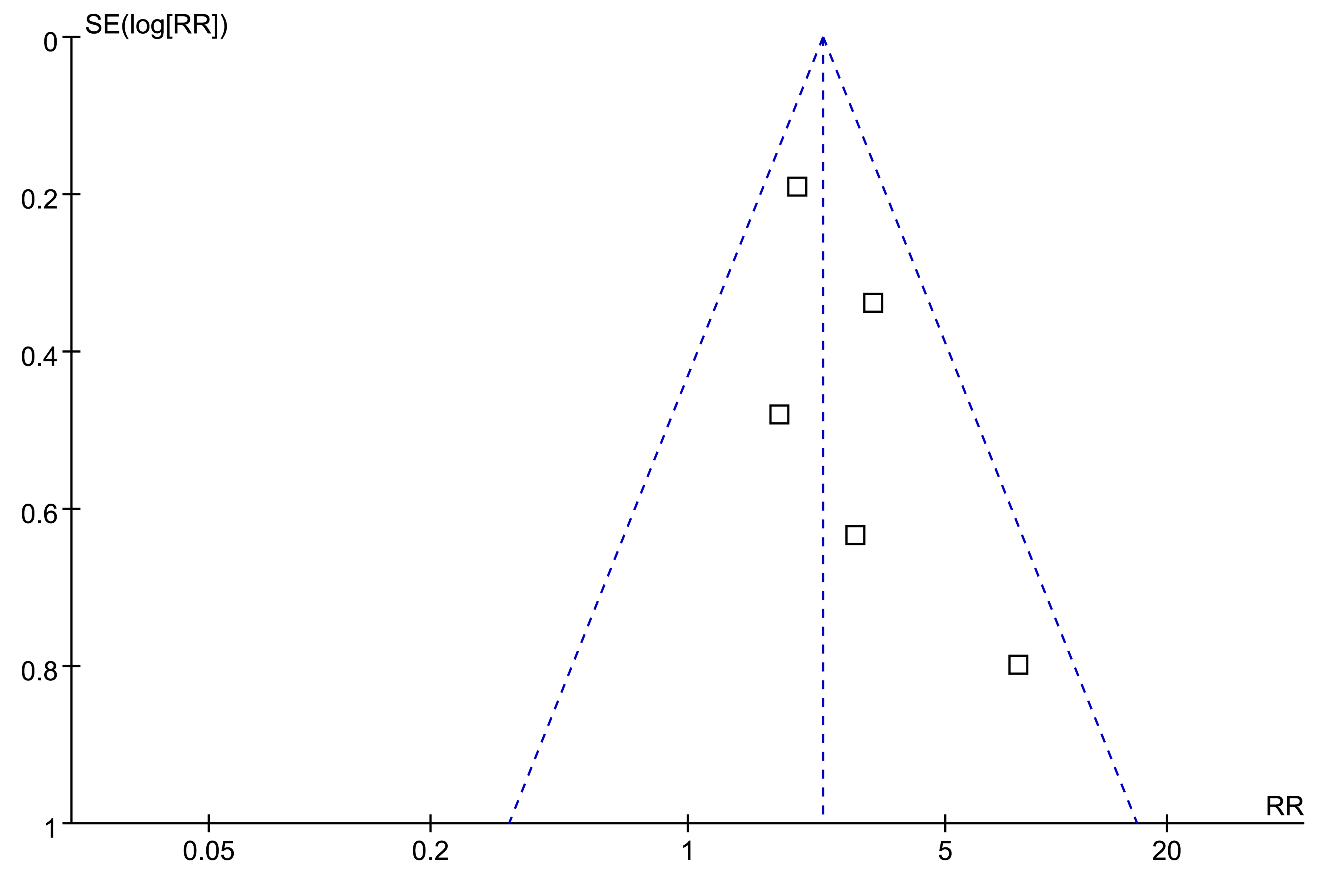


**Supplemental Figure 1** Funnel plots for the meta-analysis of the association between anxiety and AF recurrence after ablation;
